# A meta-analysis comparing tenotomy and tenodesis for treating rotator cuff tears combined with long head of the biceps tendon lesions

**DOI:** 10.1371/journal.pone.0185788

**Published:** 2017-10-09

**Authors:** Xiliang Shang, Jiwu Chen, Shiyi Chen

**Affiliations:** Department of Sports Medicine, Huashan Hospital, Fudan University, Shanghai, China; Universite de Nantes, FRANCE

## Abstract

**Purpose:**

The purpose of this meta-analysis was to assess whether there were differences in the outcomes between tenotomy and tenodesis in treating LHBT lesions combined with rotator cuff repairs.

**Methods:**

Using Medline, Embase, and Cochrane, we searched for articles comparing tenotomy and tenodesis combined with rotator cuff repair which were published before April 2016 with the terms “biceps”, “tenotomy”, “tenodesis”, and “rotator cuff”. The controlled clinical studies that met the inclusion and exclusion criteria were assessed for quality of methodology by utilizing the Coleman score.

**Results:**

On the basis of the inclusion and exclusion criteria, ten articles (903 patients) were included in this meta-analysis. The Coleman score ranged between 40 and 89 in the included studies. The results showed that the incidence of the popeye sign (OR, 2.777, P = 0.000) were higher in tenotomy group compared with tenodesis group when concomitant rotator cuff repair. Statistically significant difference in favor of tenodesis was observed for Constant score (SMD, -0.230, P = 0.025). As for the arm cramping pain, patient satisfaction, VAS score, ASES score and UCLA increased score, the strength and the range of motion, there were no significant differences between tenodesis and tenotomy of the LHBT, corresponding to the currently available results in the literature.

**Conclusions:**

Based on this meta-analysis, both tenotomy and tenodesis are effective in pain relief and function improvement in patients with repairable rotator cuff tears. No significant differences in post-operative functional outcome between tenotomy and tenodesis for the treatment of LHBT lesions were observed except for a lower Constant score and higher risk of Popeye deformity in tenotomy.

## Introduction

The long head of the biceps tendon (LHBT) lesions, including disclocation, subluxation, partial tears and tendinitis, are frequently associated with partial or complete rotator cuff tears (RCTs), particularly in elderly patients[1–8]. The most frequently used surgical managements for these lesions are tenotomy, tenodesis or debridement[9]. Just as described in detail previously[10], all of these surgical techniques have been demonstrated to be effective in alleviating pain and improving activities of daily life in patients with massive cuff tears[1, 2, 4, 7, 8]. Arthroscopic biceps tenotomy is an easy and fast procedure with less overall operating time and simplier postoperative rehabilitation [3, 11, 12] compared with tenodesis. However, it has drawbacks including the possibly deformity of the anatomic profile of the arm (“Popeye” sign) [1, 4, 8, 13], the loss of the LHB capability of stabilizing the head of the humerus [2], and the possible onset of cramping or fatigue pain [8, 14]. On the other hand, the tenodesis could theoretically avoid all these possible complications, even though a longer operating time and a longer rehabilitative procedure would be required [2, 4, 6, 15, 16]. However, which technique could result in the best patient outcome, especially between tenotomy and tenodesis, for treating patients with repairable cuff tears concomitant severe degeneration of LHBT, is still controversial.

There were numerous researches comparing the clinical outcomes of biceps tenotomy and tenodesis in treating LHBT lensions in recent years[1, 10, 17–21]. For example, De Carli et al compared the clinical, functional, and radiological results of tenotomy or tenodesis in treating LHBT degeneration with concomitant repairable rotator cuff tear [10]. They found that tenodesis did not provide more significant clinical or functional improvement than isolated tenotomy except for fewer incidences of the Popeye sign [10]. Koh et al also found that suture anchor tenodesis of the LHBT could lead to less Popeye deformity than tenotomy, while surgical times and clinical results between tenotomy and tenodesis showed no statistical difference [18].

Except from single studies, systemic reviews [14, 22] and meta-analyses were also conducted in these years to compare these two techniques [23, 24]. However, some of these studies included low-quality studies and different patient populations which might affect the results. For example, in the systermatic review and meta-analysis by Leroux et al, [23] there were 12 studies included. Of these studies, 6 were treated with tenodesis (levels 4), 3 were treated with tenotomy (levels 1 and 2) and only 3 directly compared the tenotomy and tenodesis (levels 1 and 2) [23]. Besides, in view of pathology of LHBT is most commonly encountered in the setting of RCTs, while there are only a few reports on the treatment of biceps lesions combined with RCTs and no meta-analysis compare the functional results among patients undergoing tenotomy or tenodesis of the LHBT associated with arthroscopic rotator cuff repair right now.

Therefore, in the present study, we sought to determine whether there are differences in the outcomes between tenotomy and tenodesis in treating LHBT lesions combined with RCTs by including high quality and new clinical studies, the results of which we believe would assist in treatment selection.

## Methods

### Search strategy

We performed this study following principles of the PRISMA statement (S1 Table). A comprehensive search of the published literature was performed by two independent researchers for articles that reported clinical outcomes in patients who underwent arthroscopic tenotomy or tenodesis combined with rotator cuff repair. The following databases were searched: Medline (Pubmed) (1950 to April 2016), Embase (Ovid) (1974 to April 2016), and Cochrane (1996 to April 2016). Search strategy of Embase was as follows: (‘biceps’ OR ‘tenotomy’ OR ‘tenodesis’) AND (‘rotator cuff’). This search identified 97 records. Similar searches were conducted in Pubmed and Cochrane Library Database. Relevant researches in the references of published articles were also searched. Altogether 176 records were found.

### Inclusion and exclusion criteria

Eligibility criteria for inclusion of the review were as follows: studies reporting the clinical outcomes comparing tenotomy and tenodesis combined with rotator cuff repair by using clinical or functional scoring systems. Cadaver or animal studies, biomechanical studies, literature reviews, letters to editors, expert opinion articles, case reports, or technique notes that did not report clinical outcome data were excluded.

### Outcome measurements

The following outcomes in the included studies were assessed and compared: primary outcomes including University of California at Los Angeles (UCLA) score, American Shoulder and Elbow Surgeons (ASES) score, Constant score, visual analogue scale (VAS) score, elbow flexion strength index, forearm supination strength index, range of motion and secondary outcomes including popeye deformity, cramping pain in the retracted biceps muscle and patient satisfaction.

### Data extraction

On the basis of the titles and abstracts, 2 reviewers selected relevant studies for further review. For studies included in the analysis, 2 reviewers analyzed the full articles using the previously mentioned criteria independently. The reviewers were not blinded to the author, year, and journal of publication. Disagreements between the two reviewers were resolved by consulting with a third reviewer.

Data including study characteristics (study type, patient number, and duration of follow-up), patient demographics (age, sex), and clinical outcomes (functional outcome scores, ROM, biceps deformity and cramping, average surgical time, and patient satisfaction) were extracted.

### Assessment of quality

The Coleman methodology scoring system was used to assess the methodological quality of the selected articles by two reviewers. Discrepancies were given the higher score to show the study at best. The scoring system evaluates the quality of study design, sample size, patient selection, length and completeness of follow-up, and outcome assessment [25]. The score varies between 0 and 100 with a score of 100 representing a study design which largely avoids biases, chance, and other confounding factors.

### Statistical analysis

Data analysis was performed via Stata/SE 11.0 software. According to the Cochrane recommendation, standardized mean difference (SMD) was used when the studies assessed the same outcomes with different measure ways to standardize the results of the studies to a uniform scale. For each study, SMDs with 95% confidence intervals (CIs) were calculated for continuous data (Constant score, elbow flexion strength index, forearm supination strength index, range of motion, VAS score, UCLA increased score and ASES score) and odds ratios (ORs) with 95% CIs were calculated for dichotomous data (Popeye deformity, arm cramping pain, patient satisfaction). Cho *et al*. [20] and Meraner *et al*. [26] used median and range in reporting their relevant outcomes and were unresponsive to our query for original data, so we used reliable formulas to translate median and range into mean and standard deviation (SD) for the purpose of gathering a comprehensive database [27–30]. Because these two studies had sample sizes of 41 and 29, respectively, the conversion method is defined as such that median and range/4 can give the best estimator of mean and SD, respectively, when the sample size is between 25 and 70 [31]. Heterogeneity was assessed by using *I*^*2*^. The random effects model analysis was employed. Forest plots were generated for each outcome index. *P* < 0.05 was considered as statistical significant.

## Results

### Included studies

**Fig 1:**
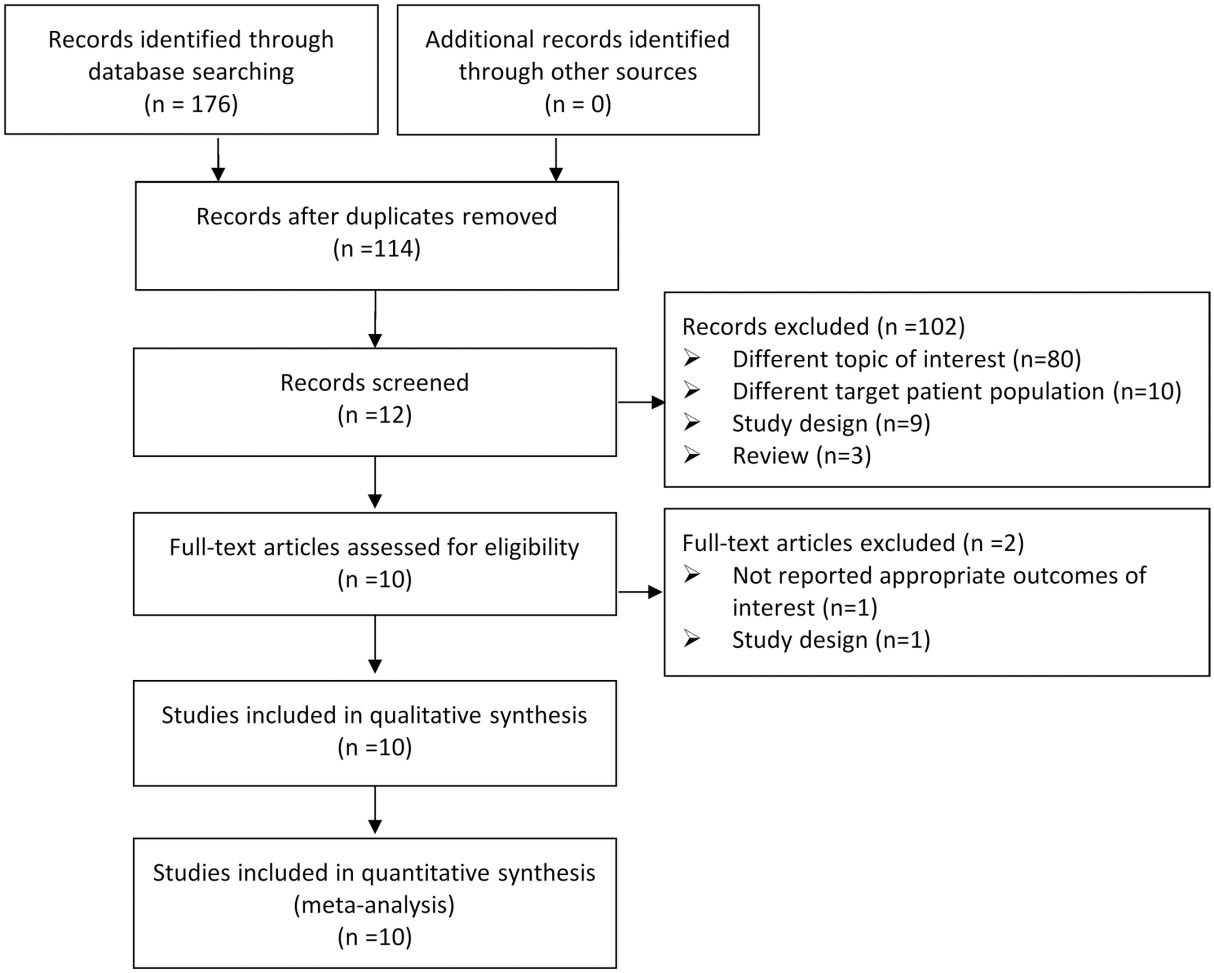
Flow chart summarizing the selection of relevant articles.

The flow chart of selecting relevant articles is shown in Fig 1. A total of 176 publications were screened out from the online database, including 73 publications from Pubmed, 97 from Embase and 6 from Cochrane. After removing the duplicates, 114 publications were left. These 114 articles were further reviewed by full-text. Finally, 10 articles reporting on 903 participants were included in this meta-analysis [10, 18–20,26, 32–36]. Among them, 361 patients were treated with tenodesis (40%) and 542 were treated with tenotomy (60%). The baseline data and characteristics of the studies are summarized in Table 1. Briefly, there were 3 randomized controlled trials, 4 cohort studies, and 3 retrospective studies. Correspondingly, level of evidence of these ten studies from level I to level IV. The quality of these studies was assessed by Coleman methodology score. As shown in Table 2, the coleman score ranged between 40 to 89 (66.3 ± 15.72). The sample sizes of most studies were larger than 60 except for Meraner *et al*. [26] and Sentürk *et al*. [36].

**Table 1:** Characteristics of the included studies.

Author	Year	Study, LoE	Participants	Intervention	Outcomes
Koh, et al.[18]	2010	Cohort study, II	84 patients (age, >55 years) with a RCT and biceps tendon lesion	41 Tenotomy 43 Tenodesis	Popeye deformity, arm cramping pain, Elbow Strength Index, ASES score and Constant score
Biz, et al.[32]	2012	Cohort study, III	252 patients, who were treated with arthroscopic surgery by the same operator for a LHB disease associated with a RCT	202 Tenotomy 20 Tenodesis-1 30 Tenodesis-2	Popeye deformity, VAS score and UCLA score
De Carli, et al.[10]	2012	Therapeutic study, II	65 patients affected by a repairable RCT along with a degenerative lesion of the LHBT	30 Tenotomy 35 Tenodesis	Popeye deformity and Constant score
Ikemoto, et al.[33]	2012	Retrospective study, III	77 patients undergoing arthroscopic repair of the rotator cuff, with LHB injuries justifying tenotomy with or without tenodesis	55 Tenotomy 22 Tenodesis	Popeye deformity, Elbow Strength Index and UCLA score
Kukkonen, et al.[34]	2013	Cohort study, II	148 consecutive shoulders operated for isolated full-thickness supraspinatus tendon tear with biceps procedure (no procedure, tenotomy, and tenodesis)	30 Tenotomy 30 Tenodesis 85 Control	Popeye deformity, arm cramping pain and Constant score
Cho, et al.[20]	2014	Cohort study, III	83 patients who underwent surgical treatment of RCTs with concomitant lesions of the LHBT	41 Tenotomy 42 Tenodesis	Popeye deformity, Constant score, VAS score, UCLA score and Range of motion
Zhang, et al.[19]	2015	Therapeutic study, I	151 patients older than 55 years of age with LHB lesions and reparable RCTs	77 Tenotomy 74 Tenodesis	Popeye deformity, arm cramping pain, Elbow Strength Index, Suspination strength index, Constant score and VAS score
Meraner, et al.[26]	2016	Retrospective case series, IV	53 patients who underwent arthroscopic double row rotator cuff reconstruction and suture bridge repair	29 Tenotomy 24 Tenodesis	Popeye deformity, arm cramping pain, Constant score and Range of motion
Oh, et al.[35]	2016	Prospective comparative study, II	86 patients who underwent arthroscopic rotator cuff repair with SLBC lesions	27 Tenotomy 31 Tenodesis 28 Debridement	Popeye deformity, arm cramping pain, Elbow Strength Index, suspination strength index, ASES score, VAS pain score, VAS satisfaction score and Range of motion
Sentürk, et al.[36]	2011	Retrospective study, IV	20 patients who were diagnosed with chronic biceps tenosynovitis	10 Tenotomy 10 Tenodesis	Popeye deformity, Constant score and UCLA score

LOE: level of evidence; ASES, American Shoulder and Elbow Surgeons score; UCLA, University of California Los Angeles score; VAS, visual analog scale

**Table 2:** Coleman methodology score and criteria.

Section score (min-max)	Mean	SD	Range
**Part A**			
1. Study size (0–10)	6.7	3.05	0–10
2. Follow-up (0–5)	1.2	1.54	0–3
3. Number of procedures (0–10)	9.4	1.84	7–10
4. Type of study (0–15)	7.0	6.32	0–15
5. Diagnostic certainty (0–5)	4.7	0.67	3–5
6. Decription of surgical technique (0–5)	4.7	0.67	3–5
7. Rehabilitation and compliance (0–10)	4.2	2.15	0–8
**Part B**			
1. Outcome criteria (0–10)	6.8	1.98	3–9
2. Outcome assessment (0–15)	9.3	2.21	5–12
3. Selection process (0–15)	12.3	3.16	7–15
Total Coleman methodology score	66.3	15.72	40–89

SD, standard division

### Meta-analysis of continuous data outcomes

The continuous data outcomes included UCLA score, ASES score, Constant score, VAS score, elbow flexion strengh index, forearm supination strength index, range of motion.

#### UCLA increased score

**Fig 2:**
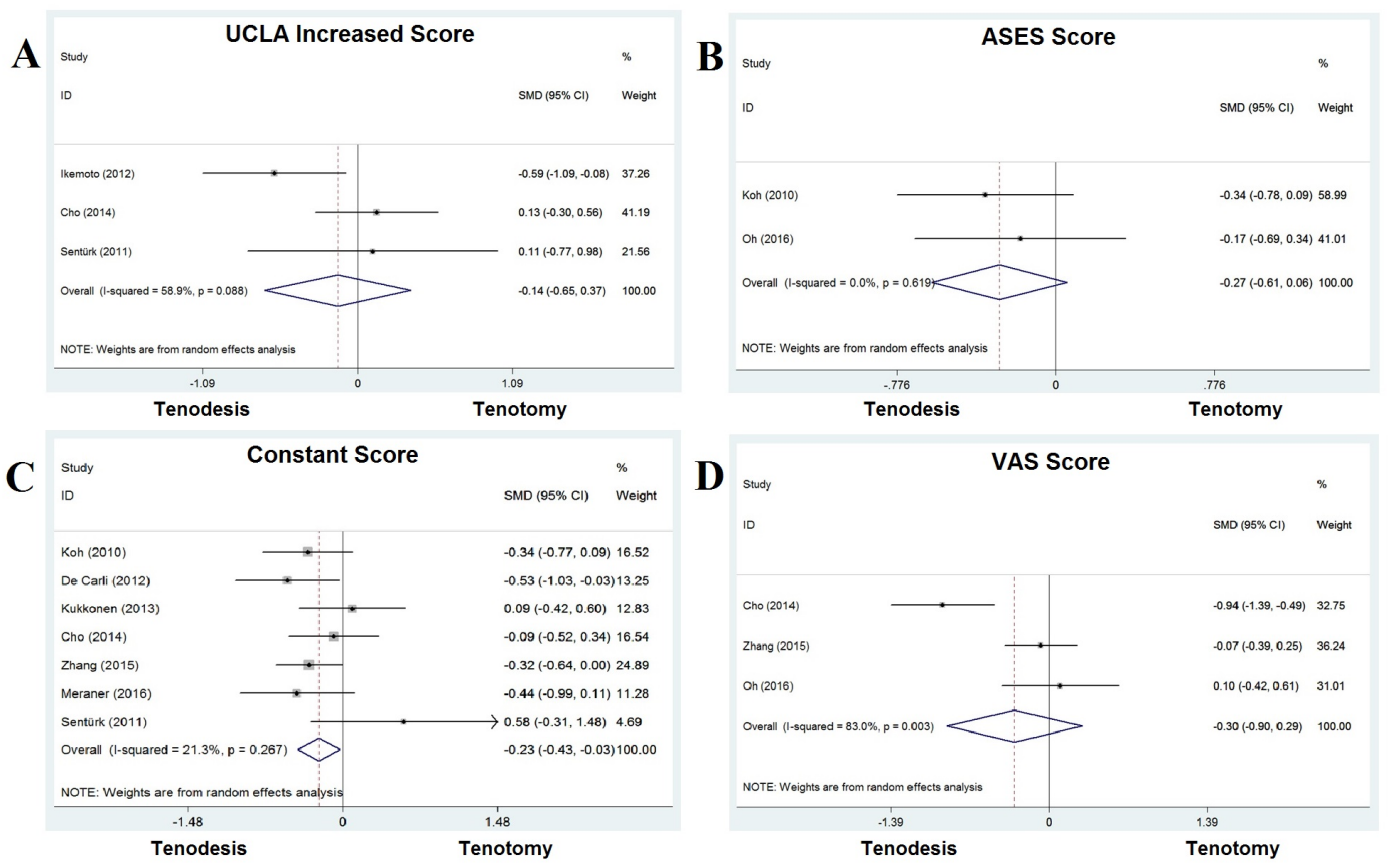
Standard differences in means for functional scores (UCLA increased score, ASES score, Constant score and VAS score) between tenodesis and tenotomy groups.

UCLA increased score was measured in three studies, in which 74 patients (20%) were treated with tenodesis and 106 patients (20%) with tenotomy. A mean difference of -0.140 [−0.654 to 0.373] was calculated, with a *P* value of 0.592, suggesting that there was no significant difference in UCLA increased score between these two groups (Fig 2A).

#### ASES score

ASES score was measured in two studies, in which 74 patients (20%) were treated with tenodesis and 68 patients (13%) with tenotomy. A mean difference of -0.274 [−0.606 to 0.057] was calculated, with a *P* value of 0.104. No significant difference was seen in ASES score between these two groups (Fig 2B).

#### Constant score

Constant score was measured in seven studies, in which 258 patients (71%) were treated with LHBT tenodesis and 258 patients (48%) with tenotomy. A mean difference of -0.230 [−0.432 to -0.029] was calculated, with a *P* value of 0.025. Constant score was significantly higher in patients with tenodesis than that in patients with tenotomy (Fig 2C).

#### VAS score

VAS score was measured in three studies, in which 147 patients (41%) were treated with tenodesis and 145 patients (27%) with tenotomy. A mean difference of -0.304 [−0.899 to 0.291] was calculated, with a *P* value of 0.316, which means there was no significant difference in VAS score between the tenodesis and tenotomy group (Fig 2D).

#### Elbow flexion strength index

**Fig 3:**
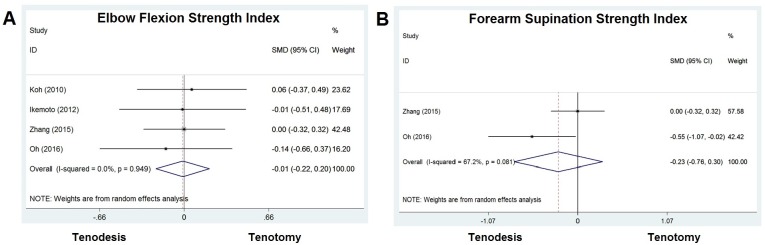
Standard differences in means for elbow flexion and forearm supination strength index between tenodesis and tenotomy groups.

Elbow flexion strength index was reported in four studies, in which 170 patients (47%) were treated with tenodesis and 200 patients (37%) with tenotomy. A mean difference of -0.012 [−0.220 to -0.196] was calculated, with a *P* value of 0.910. No significant difference was seen in elbow flexion strength index between the two groups (Fig 3A).

#### Forearm supination strength index

Forearm supination strength index was reported in two studies, in which 105 patients (29%) were treated with tenodesis and 104 patients (19%) with tenotomy. A mean difference of -0.232 [−0.763 to -0.298] was calculated, and this was no significant difference between the two groups with a *P* value of 0.391 (Fig 3B).

#### Range of motion

**Fig 4:**
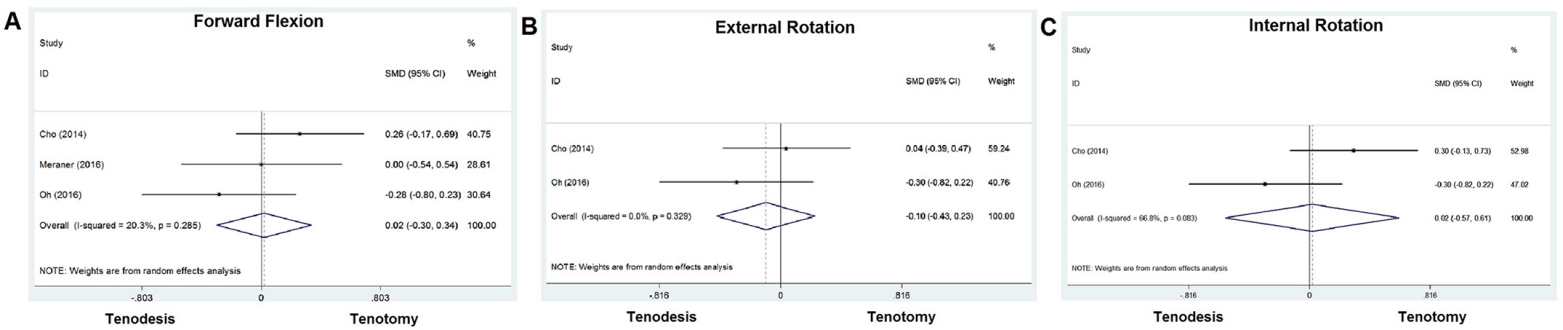
Standard differences in means for range of motion between tenodesis and tenotomy groups.

For shoulder range of motion, three indicators including forward flexion (3 studies involved), external rotation at the side (2 studies involved), and internal rotation to the back (2 studies involved) after the operation were assessed. No significant differences in forward flexion (SMD = 0.019, *P* > 0.05), external rotation (SMD = -0.098, *P* > 0.05) and internal rotation (SMD = 0.020, *P* > 0.05) were seen between tenodesis and tenotomy group (Fig 4).

### Meta-analysis of dichotomous data outcomes

The dichotomous data outcomes included popeye deformity, cramping pain in the retracted biceps muscle and patient satisfaction.

#### Popeye deformity

**Fig 5:**
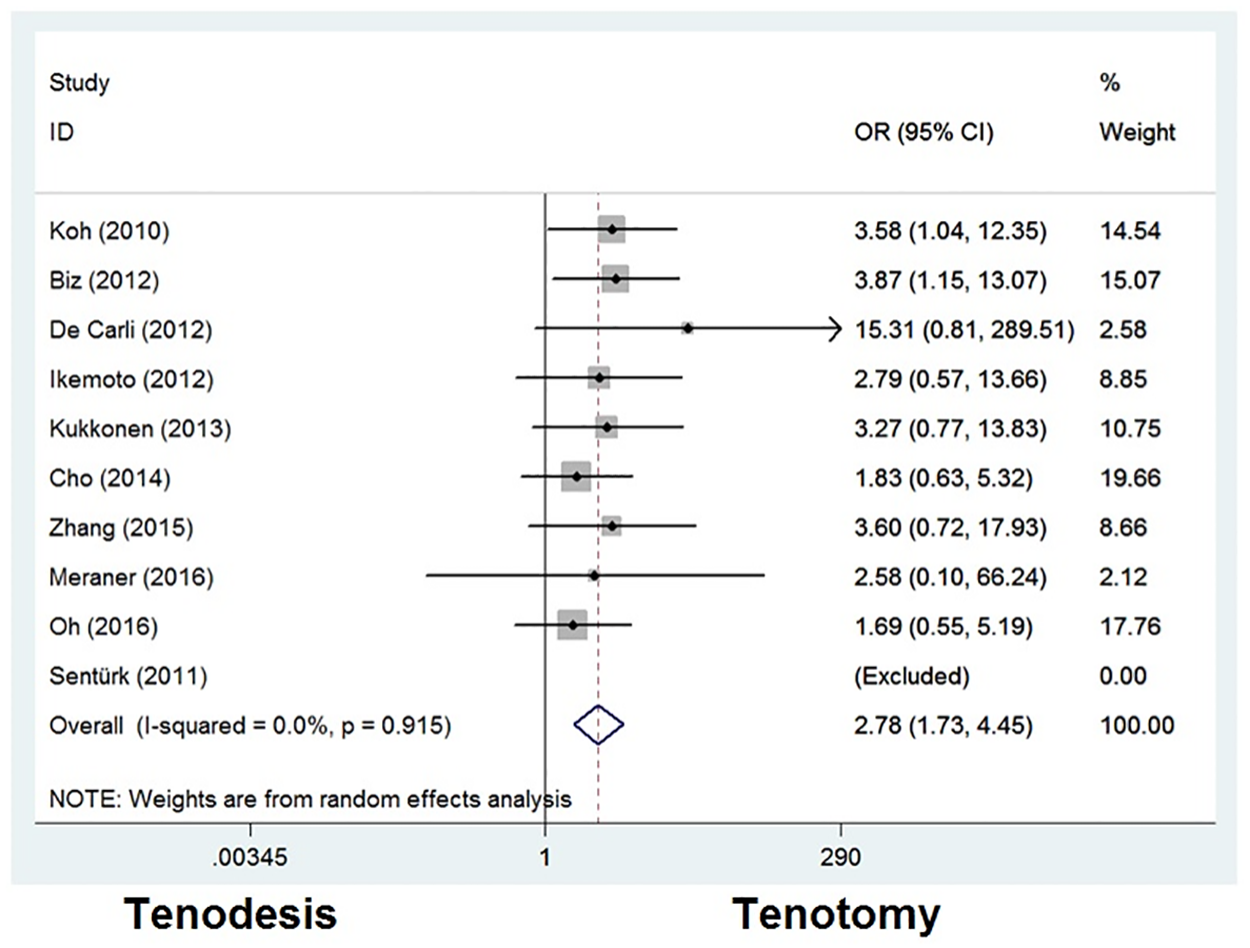
Odds ratios for popeye deformity between tenodesis and tenotomy groups.

Post-operative outcome concerning the popeye deformity in the upper arm was described in ten articles. For this outcome, the results of 351 patients (97%) treated with tenodesis and 532 patients (98%) with tenotomy of the LHBT were meta-analyzed. An odds ratio of 2.777 [1.731–4.455] was calculated in favor of tenotomy with a *P* value of <0.001 suggesting that the incidence of the popeye sign was significantly higher in patients with tenotomy than that in patients with tenodesis (Fig 5).

#### Arm cramping pain

**Fig 6:**
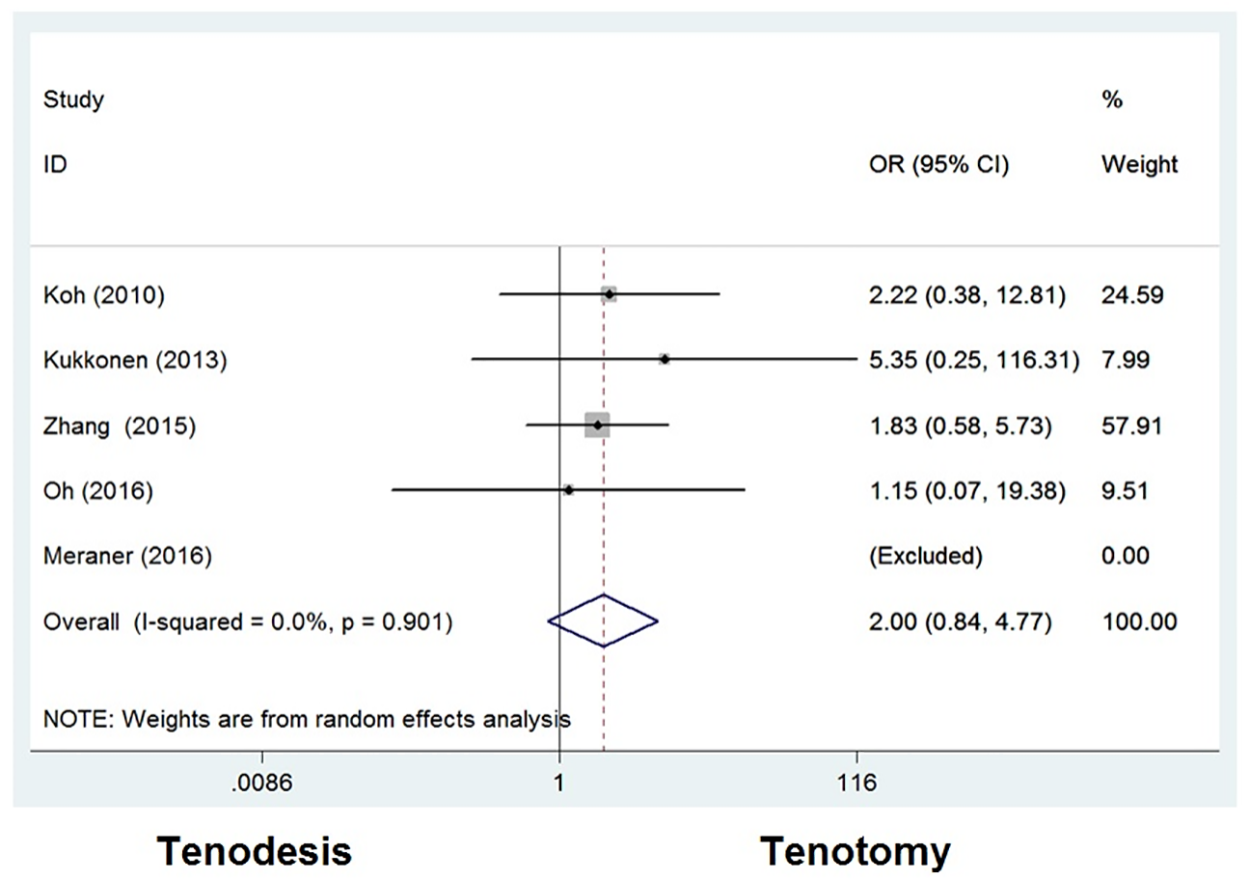
Odds ratios for arm cramping pain between tenodesis and tenotomy groups.

Cramping pain was reported in five studies, in which 202 patients (56%) were treated with tenodesis and 204 patients (38%) with tenotomy. In these five studies, Meraner *et al*. [26] did not find any arm cramping pain in tenodesis or tenotomy group. An odds ratio of 1.998 [0.837–4.769] was seen with a *P* value of 0.119 (Fig 6), suggesting that there is no significant difference in arm cramping pain between the tenodesis and tenotomy group.

#### Patient satisfaction

**Fig 7:**
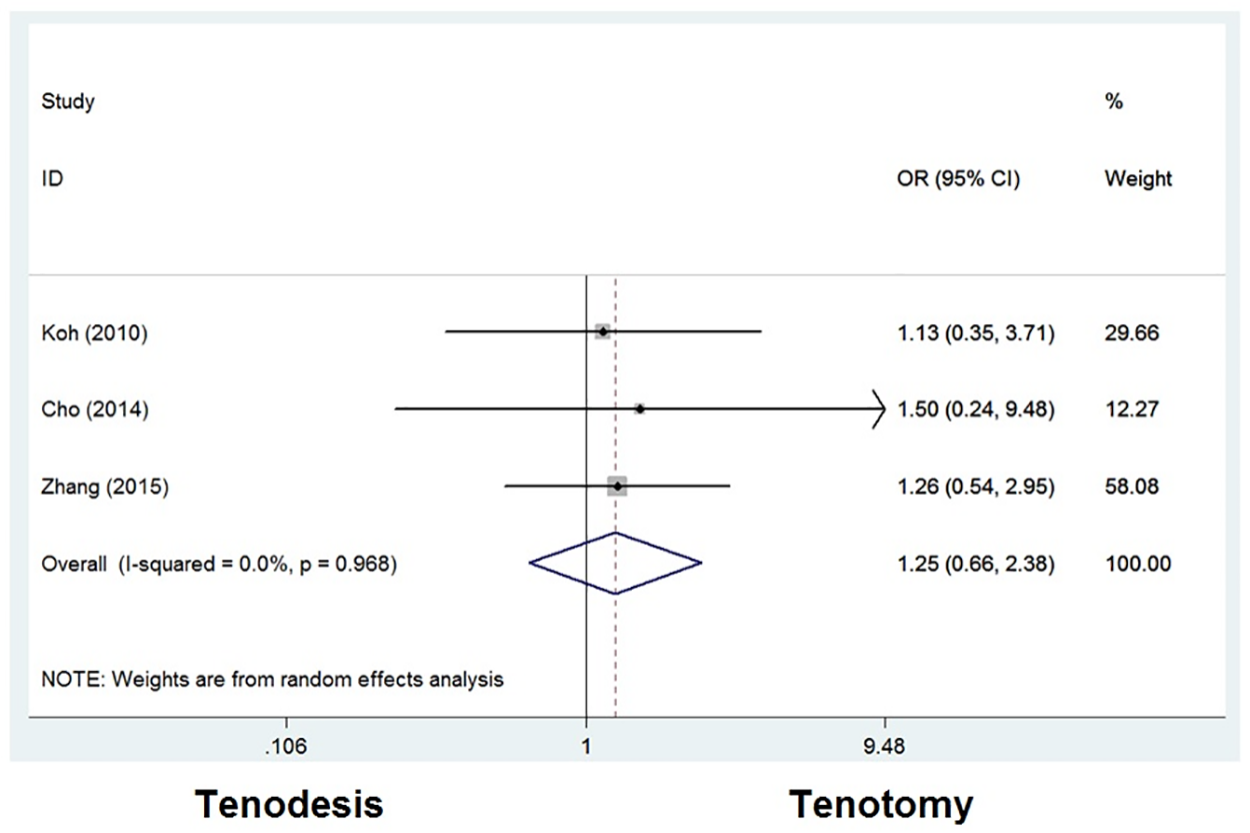
Odds ratios for patient satisfaction between tenodesis and tenotomy groups.

Patient satisfaction evaluation was reported in three studies, in which 159 patients (44%) were treated with tenodesis and 159 patients (29%) with tenotomy. An odds ratio of 1.250 [0.655–2.384] was seen with a *P* value of 0.498. No significant difference was detected in patient satisfaction between groups (Fig 7).

## Discussion

RCTs may produce more pressure and friction on the LHBT, resulting in the high risk for lesions of LHBT [37]. Accordingly, RCTs are often involved with LHBT lesions. These lesions can cause significant shoulder pain and dysfunction. They may vary in degree, ranging from minor tendinitis to a complete rupture [15]. The diagnosis for these lesions is often difficult, and it is a tough decision for surgeons to choose an optimal treatment. As described in detail previously[18], if the partial tear involves less than 25% of the tendon or the biceps lesion is invertible, a conservative method like partial debridement may be selected as a treatment [38]. However, when a tear involves more than 30% of the tendon, a subluxation, or a degenerative superior labrum anterior to posterior type II lesion is observed, only debridement or observation might not be an optimal treatment because it could result in lasting pain even after the rotator cuff surgery [39–41]. Therefore, a definitive treatment such as tenotomy or tenodesis is considered in a rotator cuff surgery.

Although tenotomy and tenodesis have both been reported to produce good clinical results, there is a constant dilemma over the preferred treatment of RCTs combined with LHBT lesions. Biceps tenotomy is a more popular operative strategy in treating tendon lesions, especially when these lesions accompanied by RCTs [39]. Supporters of biceps tenotomy advocate that it is simple, causing less surgical time in the arthroscopic setting, with simple rehabilitation, very low surgical morbidity, avoidance of implant complication, and satisfactory pain relief with minimal function impairment [19, 36]. On the other hand, advocates of biceps tenodesis believe that tenodesis can better maintain the relationship between length and tension of tendon, keep from muscle atrophy, maintain elbow flexion and supination power, avoid cramping pain, and minimize cosmetic deformities [42]. However, tenodesis takes longer surgical time than tenotomy, where a simple release is done at the junction of the biceps labrum complex. Besides, it might be complicated to perform the tenodesis by identifying the biceps tendon in the subacromial space when impingement syndrome or partial-thickness RCT is present or the cuff tear size is small.

Prior to this study, there are several meta-analysis compared the outcome of tenotomy and tenodesis, aiming to found out which of the two methods can produce better clinical and functional outcomes. For example, in the systermatic review and meta-analysis by Leroux et al, [23] they examined the clinical outcome of LHBT tenotomy or tenodesis preformed concurrently with rotator cuff repair. There were 12 studies included, however, only 3 studies directly compare tenotomy and tenodesis were used in the meta-analysis, the quality of other studies was relatively low (6 studies were levels 4) [23]. Another systematic review and meta-analysis included 7 studies compared tenotomy and tenodesis in patients diagnosed with LBHT lesions [24]. Four studies have also been included in our meta-analysis, 3 studies are not included in our meta-analysis because one reported patients with isolated LHBT lesions without RCTs, one reported type II superior labrum anterior and posterior (SLAP) lesion (not LHBT lesion) treatment associated with rotator cuff repair, one reported biceps tenotomy or tenodesis with massive irreparable rotator cuff tears[24]. A systematic review and meta-analysis performed by Gurnani et al included nine studies [43]. A total of 405 patients (62.3%) were treated with concomitant cuff tears, 176 (27.1%) with isolated LHB lesions, 34 (5.2%) with subacromial decompression, and 35 (5.4%) with undefined concomitant treatment in this review [43]. A significant limitation of this review above is the inclusion of heterogeneous patient populations that vary with respect to concomitant shoulder pathology. In our study, we sought to observe the differences in a specific patient population—-LHBT lesions combined with repairable rotator cuff tears—and statistically analyze data from only higher quality comparative studies, all other single-treatment studies and concomitant shoulder pathology don’t accord with the inclusion criteria were excluded. In view of this, we evaluated differences between tenotomy and tenodesis in treating LHBT lesions concurrent with rotator cuff repair and provide an overview on benefits and drawbacks of the respective surgical procedures. The conclusions drawn in this meta-analysis are based on a total of 903 patients (542 tenotomy and 361 tenodesis), which is more than the patients included in previous systematic review and meta-analysis by Frost *et al*.[3](420), Leroux *et al*. [23] (565), Gurnani *et al*. [43] (650) and Ge *et al*. [24](622)

The most significant finding of this meta-analysis is that the tenotomy achieves a lower Constant score and a higher risk of Popeye deformity. These results were in agreement with the reviews by Leroux et al. [23] and Ge *et al*. [24] While Gurnani *et al*. [43] reported no significant difference in Constant score between the two procedures which was inconsistent with our results. Though the Constant score was regarded an inappropriate score system for assessing isolated biceps pathology in some study, it is still the most popular primary outcome in rotator cuff surgery [44]. As for the VAS, ASES and UCLA score, arm cramping pain, patient satisfaction, the strength and the range of motion, there were no significant differences between tenodesis and tenotomy for the treatment of LHBT lesions concurrent with RCTs. These results were mostly supported by Leroux et al, [23] Gurnani *et al*. [43] and Ge *et al*. [24] However, both Gurnani *et al*. [43] and Ge *et al*. [24] found cramping pain was more frequently observed in patients treated with tenotomy, which was inconsistent with our results. This may be due to our meta-analysis included more high quality studies and more patient population.

### Limitations

One potential limitation of our meta-analysis is the number of included studies was relatively small, which may affect the statistical power for drawing powerful conclusions. Besides, the included studies were with widely ranging Coleman scores (40–89) and the inconsistent quality might bias the results and conclusions of our study. Furthermore, all the literature included in the study are English literature, so some non-English high-quality literatures which meet the inclusion requirements might be missing, resulting in biased research results. Therefore, these findings should be treated with caution and the choice of surgical methods for a patient with LHBT concomitant RCTs should take full consideration of the patient’ s situation.

## Conclusion

Tenotomy and tenodesis are effective in pain relief and functional improvement in patients with repairable RCTs. No significant differences in post-operative functional outcome between tenotomy and tenodesis for the treatment of LHBT lesions were observed except for a lower Constant score and higher risk of Popeye deformity in tenotomy. Various factors should be taken into consideration, such as ages, cost, cosmetic concern, and surgeon preferences, in order to choose an optimal surgical procedure. Because tenotomy is simpler which needs shorter operation time, and avoid implant complication, we recommend tenotomy with concomitant rotator cuff repair in older patients, with a low level of physical activity, no cosmetic concern.

## Supporting information

S1 Table PRISMA checklist. (DOC)
